# Validation of New Quantitative Lung Ultrasound Protocol and Comparison With Lung Ultrasound Score in Patients With COVID-19

**DOI:** 10.1016/j.chest.2023.07.022

**Published:** 2023-07-27

**Authors:** Micah L.A. Heldeweg, Arthur W.E. Lieveld, Amne Mousa, Luigi Pisani, Pieter R. Tuinman, ALIFE Study Group, Micah L.A. Heldeweg, Arthur W.E. Lieveld, Mark E. Haaksma, Jasper M. Smit, Amne Mousa, Peter Klompmaker, Marry R. Smit, Lieuwe D.J. Bos, Jorge E. Lopez Matta, Carlos V. Elzo Kraemer, David J. van Westerloo, Pieter R. Tuinman

**Affiliations:** a Department of Intensive Care Medicine, Amsterdam University Medical Centers, location VUmc, Amsterdam, The Netherlands; b Amsterdam Leiden IC Focused Echography, Amsterdam, The Netherlands; c Department of Intensive Care Medicine, Regional General Hospital F. Miulli, Acquaviva delle Fonti, Italy

To the Editor:

With much interest we read the article from Volpicelli et al^1^ in *CHEST* (January 2023). We congratulate the authors on developing a novel lung ultrasound protocol (LUSext) which correlates with a quantitative CT scan score to determine the extent of pulmonary lesions in patients with COVID-19. This is valuable because previously in *CHEST*, the CT severity score (CTSS) was validated for monitoring, management, and prognostication of patients with COVID-19.^2^ However, considering the novelty of LUSext, we aimed to externally validate this protocol and contribute perspectives supported by original study results.

LUSext entails coarse “sweeping the surface” for (aspecific) pulmonary abnormalities. It quantifies extension of afflicted surface, but omits information on the nature of the abnormalities. This practical approach simplifies previous protocols and may reflect total afflicted pulmonary area more accurately. This may be particularly useful in EDs, where quick and simple lung ultrasound examination may give an immediate impression of severity. On the other hand, it may also lead to loss of information because it is mechanistically and evidentially apparent that specific pathologic lung ultrasound patterns represent a distinct spectrum of severity.^3^ This difference is particularly relevant to ICU patients with extensive global pulmonary involvement that LUSext may fail to adequately differentiate. For this population, the widely validated quantitative lung ultrasound score (LUS) may be more suitable because it both evaluates extension of afflicted surface and assigns relative weights to specific pulmonary abnormalities.^4^ LUS may also be performed rapidly and has been validated for COVID-19 and non-COVID-19 ARDS and is associated with CTSS, Pao_2_/Fio_2_ ratio, extravascular lung water, length of stay, duration of ventilation, and mortality. 5, 6, 7, 8, 9, 10 There are currently no studies that demonstrate association between LUSext and patient outcomes.

The conscientious physician must now carefully consider which protocol to use for which indication, patient, and setting without information on their relative clinical utility. Therefore, the primary aim of this study was to compare LUS and LUSext for their correlations with CTSS and ventilator-free days (VFDs) in both the ICU and ED. We hypothesize that both LUS and LUSext correlate with outcome, but that LUS performs better in the ICU setting.

## Study Design and Methods

This is a post hoc analysis of four prospective observational studies in the ICU and ED of Amsterdam University Medical Center. Data collection took part from March 19, 2020, until February 1, 2022. Studies were approved by the local ethics boards, and need for informed consent was waived (Medische Ethische Toetsingscommissie VUmc 2020.011 and CMO region Arnhem-Nijmegen 2020-6372).

All clinically indicated lung ultrasound examinations in adults (aged ≥ 18 years) admitted to the ICU or ED with polymerase chain reaction-proven COVID-19 were eligible for inclusion. Ultrasound examinations were performed by investigators using a dedicated ultrasound machine with previously described methodology.^7^ Both LUS and LUSext are by definition surface samples of total pulmonary parenchyma. A 12-zone interspace examination may therefore be a representative surface sample provided that it would cover area along both anteroposterior and apical-basal axes of the lung. For LUS, investigators previously determined involvement per zone as follows^1^, ^2^, ^3^: 0 = A-line pattern, 1 = well-separated B-lines, 2 = confluent B-lines, and 3 = consolidation. For LUSext, investigators determined involvement per zone as follows^4^: 0 = A-line pattern and 1 = any abnormality covering the sampled interspace, based on previous assessment including pleural abnormalities and small consolidations. For both LUS and LUSext, to appropriately compare pulmonary involvement across protocols, a LUS index ([total LUS/total LUS achievable] × 100) was calculated. For each patient, age, sex, CTSS (normalized range 0%-100%) within 24 h, and VFDs at 90 days follow-up after examination were collected.

All data processing and analyses were performed using Python (v3.8; Jupyter Notebook) language for computing. A Spearman correlation coefficient was used to test the correlation of both LUS and LUSext with CTSS and VFDs. Separate correlations were calculated for patients in the ED and ICU to assess the protocol’s applicability in both settings.

## Results

A total of 282 examinations in 182 patients with COVID-19 (70.3% male; mean age, 62.9 ± 13.8 years) were included. Mean LUS was 31.5 ± 19.3 and 58.9 ± 32 in the ED and ICU, respectively. Mean LUSext was 66.6 ± 17.5 and 94.9 ± 12.6 in the ED and ICU, respectively. In the ICU, 85.1% of patients evaluated with LUSext had a pulmonary involvement > 90%, whereas only 9.1% of patients evaluated with LUS had a pulmonary involvement > 90%.

**Figure 1:**
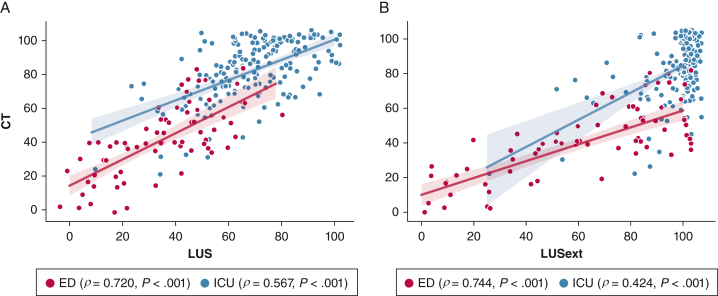
A-B, The correlation between CT severity score with LUS (A) and LUSext (B). The Spearman correlation coefficient for subpopulations with significance level is noted. The figures contain curves fitted with 95% CIs to the respective subpopulation. A jitter effect was added to improve visualization of data and avoid direct overlap of multiple examinations. LUS = lung ultrasound score; LUSext = novel lung ultrasound protocol.

The mean CTSS was 69.6 ± 25.9. A positive correlation between CTSS and LUS (ρ = 0.766, *P* < .001) and LUSext (ρ = 0.694, *P* < .001) was found (Fig 1).

**Figure 2:**
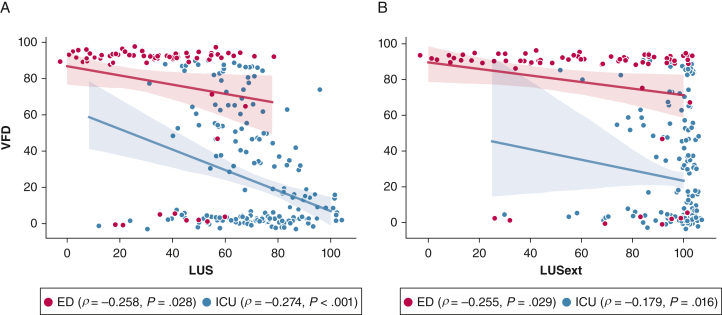
A-B, The correlation between LUS (A) and LUSext (B) with VFD at 90 d follow-up. The Spearman correlation coefficient for subpopulations with significance level is noted. The figures contain curves fitted with 95% CIs to the respective subpopulation. A jitter effect was added to improve visualization of data and avoid direct overlap of multiple examinations. LUS = lung ultrasound score; LUSext = novel lung ultrasound protocol; VFD = ventilator-free days.

Mean VFDs at 90 days was 55.5 ± 39.7. A negative correlation coefficient between VFDs and LUS (*R* = −0.562, *P* < .001) and LUSext (*R* = −0.524, *P* < .001) was found (Fig 2).

### Discussion

The results of this study to compare quantitative lung ultrasound protocols in patients with COVID-19 indicate that both LUS and LUSext are correlated with CTSS and outcome. The scatterplots also reveal an explicit ceiling effect for LUSext in many patients in the ICU, which is corroborated by lower correlation coefficients and wider 95% CIs when compared with LUS in ICU patients.

These results validate LUSext as a quantitative lung ultrasound protocol in the ED. LUSext demonstrates potential of prioritizing extent of lung injury and not pattern. However, its applicability in patients with extensive and global pulmonary affliction, as found in the ICU, is limited because of the observed ceiling effects.

LUS appears to be applicable in both settings, but has its own limitations. First, LUS requires greater pattern recognition experience, which limits its interrater reliability, particularly when differentiating between scores 1 and 2.^7^ Second, LUS does not consider the burden of other common pathologies (eg, small consolidations, pleural effusions), which certainly cause a loss of aeration. Finally, the relative weight of separate patterns must be calibrated on relevant clinical outcome parameters.

A limitation is that the methodology used to replicate LUSext (using 12-zone sampling) was an approximation of the protocol tested in a post hoc cohort. This may cause a sampling bias, even though multiple studies suggest that the examination of < 12 interspaces may adequately represent pulmonary surface affliction.^11^^,^^12^ Ultimately, these factors may limit the precision of the results, but are not likely to impact the observed data distribution. Nonetheless, we look forward to the comparison on relevant outcomes from original data by Volpicelli et al.^1^

## Interpretation

LUSext is a validated addition to the physician’s lung ultrasound armamentarium to evaluate COVID-19. However, our data suggest that LUS should continue to be the quantitative lung ultrasound protocol of choice for patients with COVID-19 in the ICU. This brings us one step closer to the optimal lung ultrasound protocol; however, several issues still need to be resolved before we can definitively settle the score.

## Financial/Nonfinancial Disclosures

None declared.
